# Dissociating Attention Effects from Categorical Perception with ERP Functional Microstates

**DOI:** 10.1371/journal.pone.0163336

**Published:** 2016-09-22

**Authors:** Benjamin Dering, David I. Donaldson

**Affiliations:** School of Natural Sciences, University of Stirling, Stirling, United Kingdom; University of Toyama, JAPAN

## Abstract

When faces appear in our visual environment we naturally attend to them, possibly to the detriment of other visual information. Evidence from behavioural studies suggests that faces capture attention because they are more salient than other types of visual stimuli, reflecting a category-dependent modulation of attention. By contrast, neuroimaging data has led to a domain-specific account of face perception that rules out the direct contribution of attention, suggesting a dedicated neural network for face perception. Here we sought to dissociate effects of attention from categorical perception using Event Related Potentials. Participants viewed physically matched face and butterfly images, with each category acting as a target stimulus during different blocks in an oddball paradigm. Using a data-driven approach based on functional microstates, we show that the locus of endogenous attention effects with ERPs occurs in the N1 time range. Earlier categorical effects were also found around the level of the P1, reflecting either an exogenous increase in attention towards face stimuli, or a putative face-selective measure. Both category and attention effects were dissociable from one another hinting at the role that faces may play in early capturing of attention before top-down control of attention is observed. Our data support the conclusion that certain object categories, in this experiment, faces, may capture attention before top-down voluntary control of attention is initiated.

## Introduction

We are able to recognise objects with only a momentary glance around our visual environment and some of these objects will capture our attention more than others. This capture of attention is guided by both bottom-up structural analysis of images and a top-down control of attention suggesting that, in a particular context, one stimulus can become most salient, for example, noticing a fire alarm in a corridor only in the event of your office burning down. Because of their social and biological importance in comparison to most other stimuli, faces are a prominent example of a visual stimulus which automatically captures attention, often to the detriment of other stimuli in the environment [1]. Faces have even been found to capture attention in visual search paradigms when they are not the explicit target [2]. These behavioural findings appear in contrast with some evidence from neuroimaging which suggests that effects of attention do not modulate early domain-specific processes in the perception of faces [3, 4]. Here, we use Event Related Potentials (ERPs) to identify the locus of attention within face perception, asking whether face-specific processes operate entirely independently of attention.

Face perception research with ERPs has focused predominantly on the N170, which is suggested to respond specifically to the presentation of a face [5–14]. The N170 is a negative peak over lateral occipital electrodes, maximal around 150–170ms post-stimulus onset. Despite the N170’s known sensitivity to many physical manipulations, such as stimulus inversion (e.g., [15]), stimulus cropping [16], and image scrambling [17], the prevailing view is that N170 face processes remain impervious to higher order cognitive effects, such as face familiarity ([11] but see [18]). Taken together, these ERP findings have been interpreted within the context of wider neuroimaging and neuropsychological accounts of face-specific processing, leading to the view that the N170 provides an index of domain specific activity for faces.

A key implication of the domain-specific view of the N170 is that the N170 should be impervious to effects of attention, either exogenous or endogenous. Exogenous attention refers to an involuntary shift in attention whereas endogenous attention reflects attention under one’s own control. Consequently, strong support for the domain-specific view of the N170 comes from studies that have manipulated attention in the context of a face perception task and failed to identify modulations of the N170 [7, 19–21]. The literature is mixed, however, with a few studies suggesting a role for attention during face-specific processing. For example, Crist et al. 2007 [8] demonstrate face-specific modulations of the N170 dependent on whether attention (endogenous, task driven) is focused on a visual stream of images that also contains faces. Sreenivasan et al., 2009 [22] also revealed task-driven attention based modulations of the N170, but only when discriminating faces was perceptually demanding. The amount of face information in the stimulus (an oval containing overlaid face and scene images at various opacities) modulated N170 when participants were focused on identifying faces. Similarly, Darque et al., 2012 [23] used an attentional blink paradigm to show that variation in the availability of attention (determined by the duration of lag between targets) leads to a modulation of the N170. Therefore, the existing evidence leaves open the possibility that so-called face specific processes reflected by the N170 may in part be driven by different forms of attention.

A second ERP effect, the P1, a positive peak in the ERP signal, occurring maximally at around 100 ms over medial occipital electrode sites, is highly sensitive to attentional demands and is modulated by selective, spatial, and non-spatial attention [24–27]. Specifically, directing attention towards a stimulus significantly increases the amplitude of the occipital P1 (and later N1) component, suggesting top-down differential processing of stimulus information at early stages in visual perception. In addition, the P1 is also modulated by changes to a number of low-level visual characteristics such as the size of stimuli [28, 29], spatial frequencies [30, 31], and luminance [26], indicating a complex interplay during the P1 range between traditionally considered low level vision and higher level cognitive control.

Like the N170, the P1 has also been proposed as evidence for early face-sensitive processing in the visual system. P1 face-sensitivity is characterized by an increase in amplitude of the component in response to faces compared to other visual stimuli [16, 32–34]. To date, however, these studies showing P1 face-sensitivity have made no claims regarding the precise nature of face-sensitive effects, whether such effects are indicative of a domain-specific network or driven by attention. Although some studies have failed to find P1 face-sensitive effects (e.g., [35, 36]), it is important to recognize that this may be driven, at least in part, by the fact that the P1 is often simply overlooked in studies of face perception ([32]; for example, [37, 38]). More recently, Rossion & Caharel 2011 [39] suggest that P1 face sensitivity may be driven by low level differences between face and other object images, perhaps explaining why Ganis, Smith, & Schendan 2012 [40] do not find a consistent P1 face-sensitive effect. Nevertheless, the P1 findings suggest a conceivable role for the P1 in face perception, albeit without verifying the underlying nature of face-sensitive processing in the P1 time-range. Given the P1’s known sensitivity to attention, it is also likely that face-sensitivity in the P1 time-range may be driven by directed attention towards faces, although to our knowledge no existing study showing P1 face effects explicitly examined the effects of attention.

Because faces capture attention more than other visual stimuli, and attention is known to modulate early visual components of the ERP signal, a plausible alternative to the domain-specific account of face perception is that face sensitivity observable in early ERP components P1 & N170 interacts with effects of attention. Attention may be driving the increased amplitudes of the P1 or N170 in comparison to other visual stimuli, reflecting the salience of face stimuli. There are many attempts to quantify object salience in basic recognition paradigms and models linking physiological responses to behavioural psychophysics (for examples see [41–43]), however, face salience is not often discussed in relation to the P1 & N170. To determine an effect of attention directed towards faces, a categorical comparison must be made between faces and other objects to estimate the magnitude of any type of attention effect on face-sensitive ERPs.

**Fig 1:**
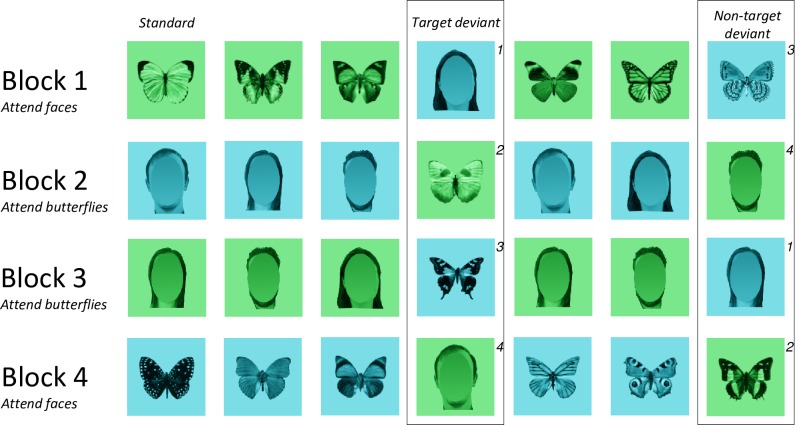
Examples of stimuli used and the experimental design (shortened for graphical purposes). All four stimulus types are utilised as standards and deviants, with their status changing between blocks. For example, if block 1 presents green butterflies as a standard, blue butterflies become the non-target deviant and blue faces are the target deviant. Note that the data we compare is always the same subset of images differing only in task demands–non-target and target deviants of the same stimulus type across the blocks, denoted here by the same numbering for targets and non-targets.

Here, we investigate the locus of attention effects in relation to face-sensitive ERPs, notably P1 & N170, by employing both a data driven analysis focusing on periods of topographic stability (functional microstates) and *a priori* predictions of ERP effects. We aimed to test a domain specific account of face perception, and a plausible face salience hypothesis suggesting that the initial perception of a face is driven by attention, using an oddball design (see **Fig 1**). This involved the presentation of a visual stimulus stream with frequent (standard) stimuli and rare (deviant) images, to quantify changes in ERPs attributable to both attention and face/butterfly stimuli (used as an object comparison). Two types of deviant oddballs were used (non-targets and targets), with non-target deviant stimuli coloured differently to standard stimuli, and target deviants marked by both their opposing colour and category to the standard. Our oddball design allows us to assess both exogenous attention shifts, observable by a comparison of standards and non-target deviants, and endogenous, task driven attention. The critical comparison in the present study is between the same image set when the focus of the task changes across blocks (i.e., when non-targets become targets), for example, in one block, all blue butterflies will be target deviant stimuli, yet in the next block they may become non-target deviants. Such a design makes unique predictions of category-sensitive effects.

**Fig 2:**
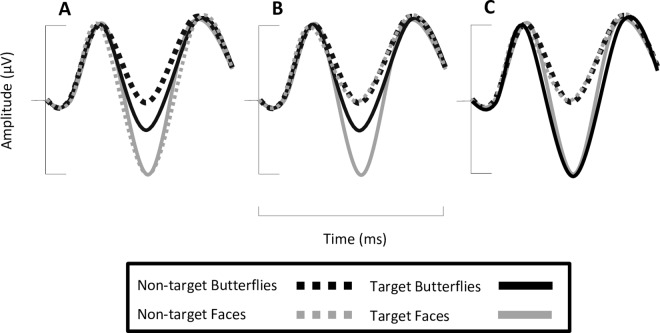
Hypothetical ERP waveforms for the four deviant conditions of the experiment that would be predicted by theories of face-sensitivity. (A) Predictions derived from a domain-specific account of ERP activity to faces; for every presentation of a face, the peak should be larger in amplitude than for other objects. Face-specificity is not affected by attention or top-down effects. (B) Predictions derived from a plausible face-salience hypothesis. The magnitude of the attention effect is increased for face stimuli compared to objects. Stimulus category and attention interact. (C) Predicts an effect of attention, but not stimulus category. Note that ERP face-sensitivity theories argue that only the N170 should be enhanced in amplitude towards faces, however these theories are applicable to any other ERP component and to the standard stimuli in the present experiment (not shown here).

In **Fig 2**, we graphically present hypothetical outcomes for the experiment based upon two accounts of ERP face-sensitivity–a domain specific and an attention-based account. Note that, while these predictions may seem focused upon the N170, since much work suggests this component forms part of a face-specific neural index [5–14], these same predictions and theoretical interpretations are equally applicable to any ERP component labelled face-sensitive. **Fig 2A** shows a prediction of ERP components if a domain-specific view of face-processing is correct. If the component is face-specific, it should be larger in amplitude for all face conditions of the experiment (including standards) and not influenced by any form of attention. **Fig 2B** suggests an interpretation of face-sensitivity that may be evident due to task-driven attention capture, i.e., that attention would elicit an increase in ERP component amplitude for faces in relation to our butterfly object comparison. **Fig 2C** gives an expected outcome if attention modulates both stimulus categories. In this scenario, ERP component amplitude would be increased by attention towards both object categories in equal proportion. Importantly, it has been claimed that the topography for the N1 to attention and the N170 for faces are fundamentally distinct (see [11]), yet scalp topographies for the attention N1 and category-sensitive N170 are rarely assessed within the same experiment. Using microstate segmentation we can gain an objective measure of topographic differences through time [44]. Our experimental design and analysis allows us to separate distinct topographies for both attention and face-sensitivity, if present in the ERP signal.

## Methods

### 2.1 Participants

Twenty-one participants all with normal or corrected-to-normal vision gave written informed consent to participate in the experiment, and were compensated for their time with course credit and £15. Two participants were removed from analyses due to poor quality data (no discernible ERP signal), leaving 19 participants (mean age = 20.8, SD = 2.2, 10 females, all right-handed). The study was approved by the ethics committee of the Psychology department at the University of Stirling.

### 2.2 Stimuli

Eighty greyscale images of both full front faces and overhead view butterflies were overlaid with a green or blue colour plate to produce four types of stimuli used in the experiment (blue/green faces and butterflies), 320 images in total (see **Fig 1:** the faces used in Fig 1 are for illustration purposes only and are masked to hide their identity). All types of stimuli generated were presented centrally on the screen, scaled to fit a standard size template, and had the same orientation in order to reduce any possible effects arising from differences in inter-stimulus perceptual variance [34, 45]. Average luminance of all images was set at 42cd/m^2^, and contrast was17.8 cd/m^2^ ±2.2. Luminance on the screen was measured using a Minolta CS-100 colorimeter and contrast values corresponded to the root mean square contrast over the whole image including background. Blue and green colour plates were overlaid on the stimuli, which were identical in terms of their saturation and value, differing only in hue (Blue plate, H = 184.94°, S = 1, V = 1; Green plate, H = 129.88°, S = 1, V = 1). Images subtended no more than 9.9 horizontal and 9.9 vertical degrees of visual angle on a black background.

### 2.3 Procedure

Participants sat in a sound attenuated room 100 cm away from a 19” TFT monitor (resolution: 1280 x 1024 pixels, 60 Hz refresh rate), calibrated to account for differences in intensity for the presentation of coloured stimuli (all green images: M = 16.8±0.75 lm, all blue images: M = 16.4±0.52 lm; measured over stimulus space on the monitor). Stimuli were psuedo-randomly presented in an oddball paradigm, split into four blocks (see **Fig 1**) such that, in each block, 640 standard stimuli were presented in sequences of 3, 4, or 5 stimuli at a time, interleaved by one of the non-target or target deviants (80 of each category in total for the block). Deviant stimuli were marked as different from standard stimuli due to their colour, e.g., if the standard is green, deviants are blue. The experiment distinguishes between two types of deviants: Target deviants that deviated in both colour and category to standards, and non-target deviants which deviate only in colour from the standards. All stimuli were presented for 400 ms and required a response, either button 1 or 5, on a 5-button response box. The target deviants required a different response from standards and non-target deviants, which took the same response, i.e., if targets required a button press of 5, standards and non-targets required a button press of 1. Responses were counterbalanced between blocks and also across participants. The inter-trial interval was randomly selected as 450, 500, or 550 ms between each stimulus presentation. Responses were counter-balanced between blocks and participants. Furthermore, participants were specifically instructed to search for target stimuli at the beginning of each block, e.g., in block 1 of Fig 1, participants were instructed to “search for faces”.

### 2.4 Event-related potentials

**Fig 3:**
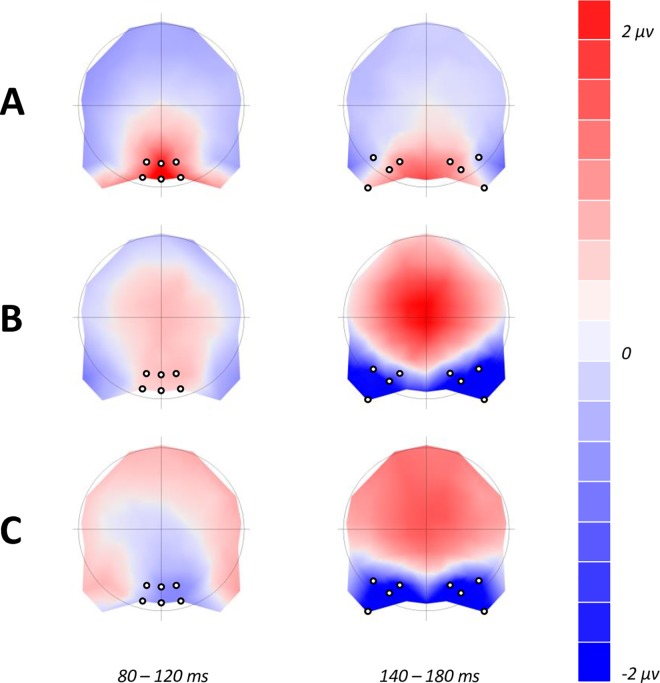
Difference topographies used to define electrodes of maximal differences between deviant conditions over the time-course of P1 (80–120 ms) & N1 (140–180 ms). (A) Differences between butterflies and faces (faces minus butterflies) regardless of deviant condition or colour. (B) Non-target minus target face stimuli, regardless of colour. (C) Non-target minus target butterfly stimuli, regardless of colour.

Scalp activity was recorded at a 1 kHz sampling rate from 64 Ag/AgCl electrodes distributed across the scalp according to the extended 10–20 system and using CZ as a reference. All impedances were kept below 5 KΩ. The electroencephalogram was filtered on-line between 0.01 and 200 Hz and off-line with a low-pass zero phase shift digital filter set to 30 Hz (48 db/octave slope). Eye blink artefacts were mathematically corrected using a model blink artefact computed for each individual based upon the method of Gratton, Coles & Donchin, 1983 [46]. Signals exceeding ±75μV in any given epoch were automatically discarded. EEG recordings were cut into epochs ranging from -100 ms to 500 ms after stimulus onset and averaged for each individual according to the experimental conditions. Grand-averages were calculated after re-referencing individual ERPs to the common average reference. The P1 was marked as the first positive peak observable in waveform data, with mean amplitudes for each condition analysed at posterior occipital electrode sites (O1, O2, OZ, POZ, PO3, & PO4) defined by visual inspection of difference topographies between 80 and 120 ms displaying maximal differences between conditions (see **Fig 3**). To investigate amplitude differences for each condition without latency effects, each mean amplitude analysis was conducted 40 ms around the peak of maximal activity of each condition (peak latency ranged from 114–119 ms). Similarly, the N170 was marked as the first negative deflection in the waveform data, analysed at electrode sites defined by visual inspection of difference topographies (sites are: CB1, CB2, P7, P8, PO5, PO6, PO7, & PO8). Mean amplitude analyses for the N170 were conducted 40 ms around the peak for each condition of the experiment (peak latency ranged from 159–170 ms). All data (behavioural, ERP, and topographical) were subjected to repeated measures ANOVAs with differing factors based upon experimental conditions and electrode sites (explained fully in the results section). ERP and topographical data reported are for correctly answered trials. Greenhouse-Geisser corrections were used and are reported where applicable. To demonstrate the magnitude of effects, partial Eta squared (ήp^2^) is also reported.

### 2.5 Topographic analysis (Microstate Segmentation)

The data from the deviant conditions were further subjected to topographic analyses (The analysis was performed using Cartool software, programmed by Denis Brunet; brainmapping.unige.ch/cartool), to look for stable patterns of scalp activity. Traditional waveform analyses characterise peaks and troughs as components of the EEG/ERP signal which are assumed to reflect different functional states of the brain, however, ERP peaks themselves do not reflect the information processing state of the brain (see [47]). Furthermore, this approach is inhibited by the choice of reference electrode, and can therefore lead to misinterpretations of data [44]. Since the configuration of the electric field at the scalp is independent of the choice of reference electrode, it can be assumed that changes in topography reflect underlying changes in neural source generators, and hence cognition [48]. Therefore, we conducted paired topographic ANOVA (TANOVA) comparisons [49] for differences between conditions to assess changes in global dissimilarity, an index of configuration divergence between two electric fields, over time [50, 51]. This analysis provides an objective measure of scalp topographies by reassigning single-subject maps to different experimental conditions–a nonparametric randomisation test over each time point and all electrodes. The outcome of the TANOVA analysis of global dissimilarity indicates, without a priori predictions, topographic differences through time. In addition, we ran a microstate segmentation analysis to delineate the underlying causes of any topographic differences which remained stable through time. Using a hierarchical cluster analysis technique, we produced, from grand averaged ERP data, a series of microstates in the form of topographic maps. The optimal number of microstates was found using a cross-validation criterion [52–54] to determine the microstates that explain the greatest amount of variance in the ERP map series. Next, we assessed the statistical validity of our segmentation by determining the amount of variance explained by each map in the ERPs of each individual by condition. Repeated measures ANOVAs were calculated on the explained variance to compare the statistical probability of each map explaining each condition, and on the GFP (global field power) assessing signal strength for each map by condition.

## Results

Our behavioural measures exhibited an expected pattern of response and reaction time (RT) for a rapid presentation task involving a rare target response switch. We subjected accuracy and reaction time data from deviant conditions to a repeated measures ANOVA model with factors of Deviant (Target, Non-Target), Category (Butterflies, Faces), and Colour (Blue, Green). We found differences in deviant accuracy such that non-target conditions were correctly identified more than target conditions [F(1,18) = 33.783, p < .05, ήp^2^ = 0.652]; on average, non-target accuracy = 93.71±4.6%, target accuracy = 68.3±21.1%. There were no differences in response accuracy for category or colour, and no significant interaction effects.

Reaction time data was filtered for all responses below 100 ms. Participants were significantly slower responding to rare deviant targets than non-targets [F(1,18) = 263.66, p < .05, ήp^2^ = 0.993]; on average, non-target RT = 270.79±39.63 ms, target RT = 442.86±38.59 ms. Furthermore, there was an interaction of deviant by category [F(1,18) = 6.507, p < .05, ήp^2^ = 0.255] such that reaction times for faces were slightly quicker than butterflies when they were targets (post hoc, P < .05), but slower when they were non-targets. No other significant differences were found in reaction time data.

### 3.1 Analysis of standards

**Fig 4:**
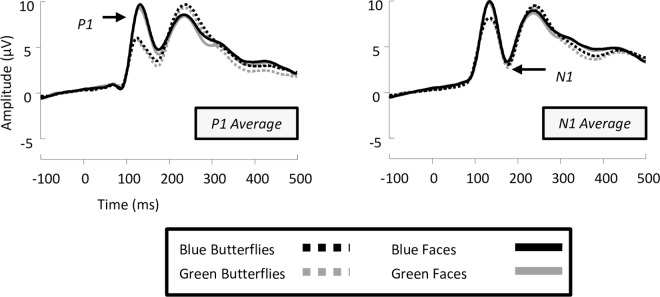
Grand averaged event-related brain potentials (ERPs) recorded from standard stimuli in the experiment. Waveforms depict an average of the electrodes used in analysis of both the P1 (O1, OZ, O2, PO3, POZ, & PO4) and N1 (CB1, P7, PO5, PO7, CB2, P8, PO6, & PO8).

ERPs for all 19 participants displayed a typical P1-N1-P2 complex for the standard experimental conditions (**Fig 4**). Repeated measures analysis of variance (ANOVA) over 6 posterior occipital electrodes defined by maximal topographic differences included 4 factors in the model: Category (Butterfly, Face), Colour (Blue, Green), Location (Occipital, Parietal-occipital) & Electrode site (O1, OZ, O2, vs. PO3, POZ, PO4). This revealed a main effect of category on P1 mean amplitude [F(1,18) = 45.778, p < .05, ήp^2^ = 0.718], showing that the P1 elicited by faces was significantly larger than the P1 elicited by butterflies. Furthermore, colour did not influence amplitude differences found in the P1 range [F(1,18) = 2.097, p>.05]. Category interacted with location [F(1,18) = 14.571, p < .05, ήp^2^ = 0.447], demonstrating a larger face-effect present in occipital compared to parietal-occipital sites. No other effects in the P1 range reached significance, and there were no significant latency differences between conditions.

For the N170, our repeated measures ANOVA model contained four factors: Category (Butterfly, Face), Colour (Blue, Green), Hemisphere (Left, Right) & Electrode site (CB1, P7, PO5, PO7, vs. CB2, P8, PO6, PO8). We found no main effects of category [F(1,18) = 2.777, p>.05], colour [F(1,18) = 1.359, p>.05] or hemisphere [F(1,18) = 1.314, p>.05]. Apart from overall changes in mean amplitude across electrode sites, we found no significant differences driven by experimental conditions in the N170 range for amplitude or latency measures.

### 3.2 Analysis of P1 deviants

**Fig 5:**
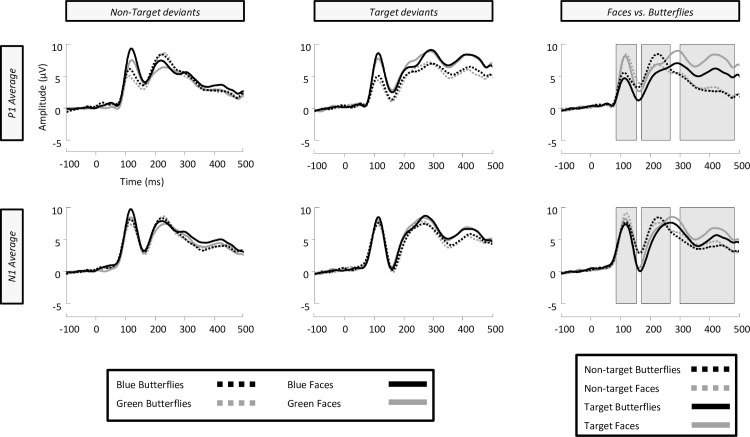
Grand averaged event-related brain potentials (ERPs) from left to right of non-target deviants, target deviants and a comparison of faces vs. butterflies regardless of colour. All waveforms depict an average of the electrodes used in analysis of both the P1 (O1, OZ, O2, PO3, POZ, & PO4) and N1 (CB1, P7, PO5, PO7, CB2, P8, PO6, & PO8). Grey shading in the faces vs. butterflies comparison shows periods of stable topographic differences indicated by paired TANOVA analysis of butterfly vs. face stimuli.

The analysis of P1 deviants involved a repeated measures ANOVA model including five factors: Deviant (Non-target, Target), Category (Butterfly, Face), Colour (Blue, Green), Location (Occipital, Parietal-occipital) & Electrode site (O1, OZ, O2, vs. PO3, POZ, PO4). ERPs for all 19 participants showed a typical P1-N1-P2 pattern for the deviant experimental conditions (**Fig 5**). We found a main effect of category [F(1,18) = 29.548, p < .05, ήp^2^ = 0.621], such that faces produced larger P1 amplitudes than butterflies, replicating the pattern of category sensitivity found for standards. Non-target deviants increased the amplitude of the P1 in relation to target deviants [F(1,18) = 12.767, p < .05, ήp^2^ = 0.415], with greater P1 amplitudes observed at occipital locations [F(1,18) = 21.959, p < .05, ήp^2^ = 0.550]. Critically, the effect of deviant was not influenced by stimulus category [F(1,18) = 0.783, p>.05]. There was a main effect of colour in the P1 range [F(1,18) = 26.872, p < .05, ήp^2^ = 0.599], with blue stimuli producing greater P1 amplitudes than green, which was evident primarily in the non-target deviants [F(1,18) = 7.048, p < .05, ήp^2^ = 0.281]. Furthermore, the P1 was also found to be larger in amplitude at occipital locations in contrast to parietal-occipital sites [F(1,18) = 11.086, p < .05, ήp^2^ = 0.381], driven by the larger amplitudes for blue, and face conditions observed in occipital locations [F(1,18) = 6.844, p < .05, ήp^2^ = 0.261]. Further planned comparisons showed that, interestingly, non-target deviants increased the amplitude of the P1 component for butterflies [F(1,18) = 12.594, p < .05, ήp^2^ = 0.412], however, P1 amplitudes for faces were not affected by the type of deviant [F(1,18) = 2.254, p>.05]. Moreover, the magnitude of the category effect in the P1 was roughly equal in both the non-target and target deviant conditions [F’s(1,18)≥24.315, p’s < .05, ήp^2^’s ≥ 0.575] (see **Fig 5.**–effect sizes are 0.575 & 0.588 respectively).

Finally, the latency of the P1 peak was significantly delayed for non-target deviant stimuli in comparison to target deviants [F(1,18) = 5.398, p < .05, ήp^2^ = 0.231], however, this difference is in the order of a few milliseconds (approx. 2.11 ms). No other comparisons of P1 peak latency approached significance.

### 3.3 Analysis of N170 deviants

The analysis of the N170 entailed an ANOVA model with five factors: Deviant (Non-target, Target), Category (Butterfly, Face), Colour (Blue, Green), Hemisphere (Left, Right) & Electrode site (CB1, P7, PO5, PO7, vs. CB2, P8, PO6, PO8). Firstly, there was no main effect of colour in the N170 range [F(1,18) = 1.241, p>.05], or any interactions with this factor. A main effect of deviant was found [F(1,18) = 54.107, p < .05, ήp^2^ = 0.75], demonstrating that target deviants produced larger N170 amplitudes than non-target deviant conditions. Further planned comparisons revealed a significant increase in N170 amplitude for face stimuli when they were targets versus non-targets [F(1,18) = 33.846, p < .05, ήp^2^ = 0.653], and this pattern was also found for butterfly stimuli [F(1,18) = 47.651, p < .05, ήp^2^ = 0.726]. While it appears that the magnitude of this effect was larger for butterflies than faces, as can be observed in **Fig 5**, there were no differences between the size of these effects in the target deviant condition [F(1,18) = 0.97, p>.05]. Indeed, there was no significant main effect of category [F(1,18) = 1.23, p>.05], suggesting that faces and butterflies did not produce different N170 amplitudes, and like the P1, the deviant factor did not interact with category [F(1,18) = 0.202, p>.05]. Interactions with electrode sites suggested that only a subset of electrodes (PO5 & PO6 being the strongest) trended towards a larger N170 for butterflies compared to faces, increased further by the target deviant condition [F (1.184,32.645) = 5.268, p < .05, ήp^2^ = 0.226]. No individual electrode comparisons displayed a larger N170 for faces. In addition, there were no significant differences between N170 peak latencies for any conditions.

### 3.4 Further planned ERP comparisons

We further compared the amplitude of the P1 & N170 for all conditions, to investigate ERP amplitude for standards in relation to both types of deviants. For the analysis of P1, the ANOVA model is the same as the analysis of P1 deviants except with the addition of standards. This analysis revealed a main effect of condition (standard, non-target, target) on P1 amplitude, with standards producing the largest P1 in comparison to non-target and target deviants respectively [F (1.789,32.211) = 22.287, p < .05, ήp^2^ = 0.553]; post hoc mean differences were all significant between the three conditions (P’s < .05). For the N170, the ANOVA model is also the same as the analysis of N170 deviants except with the addition of standards. Post-hoc analysis of the differences between standards, non-target deviants & target deviants [F (1.206,46.636) = 53.198, p < .05, ήp^2^ = 0.747] revealed no significant N170 mean amplitude differences between standards and non-target deviants. A significant N170 mean amplitude difference between target stimuli and both non-targets and standards was revealed (p’s < .05). In sum, standard stimuli evoked the largest P1 component, whereas greater changes in N170 amplitude were elicited by target stimuli.

Finally, we conducted an analysis of N170 amplitude to check for any potential changes that may have occurred in response to stimuli that were previously standards becoming targets, i.e., that lack of N170 amplitude differences may be driven in part by sequence effects. Because of the random presentation of blocks in the experiment, we analysed standards in the first block of the experiment and compared them to when they became targets (see **Fig 1**). We contrasted this with targets from the first block and when they subsequently became standards. Therefore our ANOVA model involved factors of Presentation Order (first block, after first block), Condition (standard, target), Hemisphere (Left, Right), & Electrode site (CB1, P7, PO5, PO7, vs. CB2, P8, PO6, PO8). A potential sequence effect would be present if there was a suppression of N170 amplitude from presentation order interacting with condition. We found no effect of presentation order [F (1,18) = 0.001, p>.05] and critically no interaction between order and condition [F (1,18) = 0.228, p>.05]. The order of block presentation, and therefore sequence of standards then becoming targets, did not affect ERP component amplitude.

### 3.5 Topographical analysis of deviants

**Fig 6:**
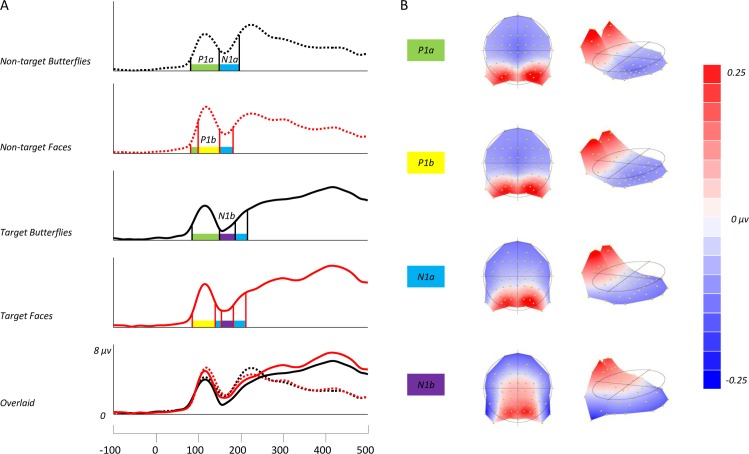
(A) Global field power (GFP) waveforms for the four deviant conditions of the experiment, with microstates encompassing the P1 & N1 range shown. Overlaid waveforms show differences in GFP for all four conditions. (B) Topographic microstates derived from the segmentation procedure for three maps which best fit the individual subject data. Our segmentation found category differences in the P1 range, but not the N1.

In order to map the time course of ERP differences that remain stable over time, we conducted a paired TANOVA comparison between all deviant butterflies and deviant faces regardless of the manipulation of colour. This highlighted three significant windows of topographic dissimilarity (see **Fig 5**for TANOVA overlaid on waveforms), which appeared to encompass the P1 and early N170 range (85–155 ms), late N170 and P2 range (171–268 ms), and a later P3 window (303–485 ms). Interestingly, this analysis showed no differences in topography at the maximum amplitude of N170, suggesting that topographies between faces and butterflies in this time period (156–170 ms) were indistinguishable. To further qualify these TANOVA differences and their relation to the experimental conditions we ran a microstate segmentation analysis (**Fig 6**). This procedure identified two maps in the P1 range, which we have labelled P1a and P1b, and subjected them to a repeated measures ANOVA of Map (P1a, P1b), Deviant (Non-target, Target), and Category (butterflies, faces). Both P1a and P1b explained a greater proportion of variance for non-target compared to target conditions [F(1,18) = 30.186, p < .05, ήp^2^ = 0.626]. Also, a significant interaction between deviant and category was found, suggesting that non-target faces are better explained than all other conditions by both maps [F(1,18) = 7.231, p < .05, ήp^2^ = 0.287]. Finally, a category by map interaction [F(1,18) = 5.334, p = .033, ήp^2^ = 0.229] demonstrates a much better fit for P1b to faces than butterflies, and P1a to butterflies compared to faces. In the case of the N1, we found two maps which we labelled N1a and N1b (**Fig 6**). These were subjected to a repeated measures ANOVA of Map (N1a, N1b), Deviant (Non-target, Target), and Category (butterflies, faces). The N1a map explained a greater proportion of variance across all conditions than the N1b map [F(1,18) = 10.313, p < .05, ήp^2^ = 0.364], with the presence of the N1b map emerging only in target conditions [F(1,18) = 3.623, p = .073, ήp^2^ = 0.168]. No main effect of category was found for either N1 map [F(1,18) = 0.133, p>.05], but there was a trending deviant by category interaction [F(1,18) = 4.298, p = .053, ήp^2^ = 0.193], suggesting that both N1 maps best fitted non-target butterflies compared to targets, and conversely target faces compared to non-targets.

## Discussion

We aimed to test if ERP correlates of face-perception were impervious to effects of attention, which could support a domain-specific view of face perception, or if attention was a driving factor in determining face-sensitive ERPs. Firstly, we replicated previous findings of P1 face-sensitivity in ERPs, supported by topographic differences between faces and butterflies (see also [16, 34]), yet found no topographic effects of task-driven attention at the P1. Since the P1 findings are similar to the proposed outcomes given in **Fig 2A**, these results could support a domain-specific account of P1 face-sensitivity, which will be examined further below. Critically, we found that the N170 showed no signs of category-sensitivity, no topographic differences encompassing the N170 peak maximum, or topographic distribution that implied a category effect for faces. The pattern of results at N170 follows the predicted outcome given in **Fig 2C**, that task-driven attention affects both object categories equally. In line with previous studies, attention significantly modulated our N170 component, supported by a distinct topography focused around the N170 present for target deviant conditions only [25, 55]. We have previously challenged the notion that the N170 is a face-selective measure and highlighted methodological reasons for this view [16, 34, 45, 56], and the present data further suggests that any face-sensitivity observed in the N170 range is not a product of increased salience of faces [57, 58].

Theories of face processing applied to ERPs have been built around the finding that the N170 is largest in amplitude to face stimuli in comparison to other objects. Here, when comparing target deviant stimuli no electrode showed larger N170 amplitudes for faces, in fact the trend of the waveforms was larger for butterflies at the N170. We suggested an alternative account of ERP face-sensitivity built around behavioural evidence which shows attention capture by faces and attention affecting early visual ERPs. However, in the present design we failed to find an N170 effect which would indicate that faces may capture more attention than our comparison butterfly stimuli. When attention was directed towards faces, the magnitude of the N170 increase observed was similar to the increase in N170 seen for attention directed towards butterflies, vetoing strict modular processing in the N170 range for faces and objects (like butterflies). Our microstate analysis confirms this ERP finding. Carmel & Bentin 2002 [7] argued that while it is acceptable that attention may modulate the N170 observed for non-face objects, there is no data to suggest that this would be the case for faces. Since this publication, there have been a few instances suggesting that task-driven attention modulates the N170 for faces (e.g., [8, 22, 23]), yet our ERP and microstate findings show that the N170 is modulated by task-driven attention equally for both faces and other objects.

It is worth reiterating the distinctions between different forms of attention, with task-driven endogenous attention (under one’s own control) resulting in N170 amplitude differences shown both here and elsewhere [8, 22, 23], compared to exogenous attention, an involuntary shift of attention, outside of one’s control. As of yet, an exogenous attention effect for faces has not been shown in ERPs. Our oddball design, using both an ignored and target deviant, allowed us to compare conditions when the focus of attention was directed away from both faces and butterflies–in these instances the N170 also showed no category-specific differences for faces. Furthermore, our oddball design did not elicit a visual mismatch negativity (vMMN) component in the N170 range, an effect of deviant minus standard stimuli resulting from automatic prediction error. Our standard stimulus changed between trials meaning that stimulus repetition, and therefore prediction at the neural level, could not take place (see [59]). We believe that the task demand to focus specifically on finding targets may have also contributed to the lack of a vMMN effect, or in other terms, an involuntary shift of attention towards faces.

Do these findings mean that no face-sensitivity can be observed in the N170 range? The present study focused on object categorisation using a simple category discrimination task, the rationale being not to test later stages in face/object perception such as discrimination between individual items. The nature of our design means that inferences about the stages of face-processing in the N170 range beyond the level of categorisation are not valid at this time, yet there is much evidence in support of later stages in face perception modulating N170 amplitude [6, 18, 38, 60]. What if a significant N170 amplitude increase were found for categories of objects other than faces? The N170 face effect is easily explained from a number of psychological perspectives suggesting why faces are special, for example, from an evolutionary perspective (sexual selection) or social psychology (important for communication), yet N170 amplitude increases for objects (e.g., [45]) are not readily explained in these terms. To summarise, the pattern of ERP (and microstate) data observed in the N170 time range does not follow the predictions derived from current theories of N170 face-sensitivity (**Fig 2**), nor is the N170 modulated by face salience.

Does our P1 component reflect activity of a domain-specific face network? Since we have consistently observed across a number of studies [16, 34, 45, 56] amplitude increases in the P1 range for faces compared to other object stimuli, one may be forgiven for making this exact claim. First of all, it is unlikely that the P1 reflects activity of a domain-specific face-network given the known sensitivity of the P1 to top-down directed attention and attention during visual search [24–27]. It is worth noting that no topographic effect of attention was found in the P1 range for faces or objects, yet our design did not test P1 domain-specificity in the context of a visual search paradigm manipulating spatial attention. Further assessment of spatial attention and P1 category sensitive effects would need to be made to examine the possibility of domain-specificity in the P1 range. Alternatively, it is possible that face-expertise could readily explain ERP results that do not support a domain-specific view. Undeniably, object expertise has been shown to modulate components of the ERP signal, although at the N170, not the P1 (e.g., [61]). However, we have no way to quantify face expertise or indeed decipher the precise nature of face-expertise. The most convincing work demonstrating effects of visual expertise have not used faces [62–64].

From our data, we may tentatively suggest that the P1 category effect reflects the initial categorisation of the visual stimulus as a face, since this was the precise nature of our task. We make no claims regarding domain-specificity in the P1 range, this requires further examination, especially since there are studies which do not report finding a P1 face-sensitive effect (e.g., [35, 36]). While ERPs displayed small attention effects on the P1, our topographical analysis confirmed that object category, not attention, drove the differences in scalp distribution for the P1 time-range. Note that one does not need to be an expert with faces or other visual stimuli to be able to categorise; categorisation itself, like the development of expertise, can be subjective (see [65]). However, it is more plausible that the face configuration contains set parameters that are easily distinguishable in low-level properties of the image (for example, see [66]). In the present experiment, face and butterfly stimuli were matched roughly in terms of size and average luminance, yet this is not to say that local luminance or contrast profiles would not influence the response profile of the P1 component (see [39] for an example of larger P1 to category specific phase-scrambled images). We suppose that the set configuration of localised features, and therefore low-level properties, in face images may be driving early face-categorisation, reflected here in the P1.

Note that ERP amplitude increases/decreases for faces should not be indicative of face processing/lack of face processing in either the P1, N170, or later time-ranges. For example, we have shown previously that when difficulties in categorisation tasks occur, the P1 is increased in amplitude ([16], experiment 2). Here we also found differences in the P1 range between coloured stimuli for non-target deviant conditions, yet critically, this was not the case for the standard stimuli highlighting that stimulus control was not driving this effect. Our coloured stimuli differed only in terms of hue, therefore the increased P1 for the colour blue observable for non-target deviants is most likely to reflect salience of colour. That is, when not the focus of the task, blue deviant stimuli appeared more salient than green deviant stimuli. No effect of colour was found in the N170 range for standards or deviants, suggesting that effects of (exogenous) attention capture are observable at an earlier stage in perception than task-driven (endogenous) attention in the N170 (see [67]).

It is worth highlighting a caveat here that poor accuracy to target stimuli in comparison to standards and non-targets was present in the study. One suggestion is that reduced accuracy to targets may be due to feature integration, combining both aspects of colour and category when deciding to make a response. However, we note that the task required participants only to search for faces or butterflies, i.e., a change in category for target stimuli, meaning feature integration is not critical for completing the task. More likely, we believe that reduced target accuracy is due to the repetitive nature of the task. Participants were required to make a response for every stimulus, with the most frequent response being made for standards and non-target stimuli (87.5% of trials in a block). When participants were required to switch their response for target stimuli, more errors were naturally expected. Furthermore, because of the rapid presentation of images (400 ms per image with an average ISI of 500 ms), participants had a limited time window to respond before the next trial occurred. It is therefore possible that the absence of a category effect in the N170 range may be in part due to reduced accuracy for participants on the task; however, we note that category-sensitive differences in the N170 range are not always observed during categorisation tasks with high accuracy (see [16, 45]).

To conclude, our results suggest that the effects of task driven attention and category sensitivity are independent of one another. Early face-categorisation around the P1 may be separable from task driven attention found later in the N170, which has implications for current theories of face-processing with ERPs. Mapping the time-course of early face perception has traditionally focused exclusively on the N170 component, yet a domain-specific account of N170 effects is not consistent with the profile of N170 data reported here. Neither should we accept the idea that the P1 is domain-specific, even though the pattern of P1 and microstate segmentation data fits with the suggestion that category sensitivity occurs pre-attentively. It is entirely plausible that early P1 face-sensitivity may reflect salience towards faces. We stress that amplitude differences in the N170 range are not of singular importance for the study of face perception with ERPs, rather an emphasis should be placed on scalp topographies that remain stable (microstate segmentation analysis is one method which allows us to determine stable topographies) during early visual perception (see also [56]). The N170 we found reflected purely the allocation of attention to target stimuli in this experiment, regardless of category. Future studies should focus upon the localised low-level properties of face images that remain constant in contrast to other objects to elucidate how face-categorisation interacts with other forms of attention, and if face salience may be driving early face categorisation.
